# Insights into *Campylobacter jejuni* colonization and enteritis using a novel infant rabbit model

**DOI:** 10.1038/srep28737

**Published:** 2016-06-30

**Authors:** Yuwei Shang, Fangzhe Ren, Zhaojun Song, Qiuchun Li, Xiaohui Zhou, Xiaobo Wang, Zhonglan Xu, Guangyu Bao, Ting Wan, Tianyao Lei, Nan Wang, Xin-an Jiao, Jinlin Huang

**Affiliations:** 1 Jiangsu Key Lab of Zoonosis, Jiangsu Co-Innovation Center for Prevention and Control of Important Animal Infectious Diseases and Zoonoses, Yangzhou University, Yangzhou 225009, China; 2 Department of Pathobiology and Veterinary Science, the University of Connecticut, Storrs 06269, United States of America; 3 College of Veterinary Medicine, Yangzhou University, Yangzhou 225009, China; 4 Yangzhou Maternity and Infant Hospital, Yangzhou 225001, China; 5 Yangzhou First People’s Hospital, Yangzhou 225004, China

## Abstract

A lack of relevant disease models for *Campylobacter jejuni* has long been an obstacle to research into this common enteric pathogen. Here we used an infant rabbit to study *C. jejuni* infection, which enables us to define several previously unknown but key features of the organism. *C. jejuni* is capable of systemic invasion in the rabbit, and developed a diarrhea symptom that mimicked that observed in many human campylobacteriosis. The large intestine was the most consistently colonized site and produced intestinal inflammation, where specific cytokines were induced. Genes preferentially expressed during *C. jejuni* infection were screened, and *acs*, *cj1385*, *cj0259* seem to be responsible for *C. jejuni* invasion. Our results demonstrates that the infant rabbit can be used as an alternative experimental model for the study of diarrheagenic *Campylobacter* species and will be useful in exploring the pathogenesis of other related pathogens.

*Campylobacter jejuni* is a leading cause of food-borne bacterial enteritis in both industrialized and developing nations, resulting in high levels of morbidity and economic loss^1^,^2^. The Centers for Disease Control and Prevention USA estimates that over 1.3 million people are infected with *C. jejuni* every year in the United States, resulting in 76 deaths annually^3^. In developing countries, the incidence of *Campylobacter* enteritis is as high as 0.4 episodes per child per year^4^. *C. jejuni* readily colonizes a wide variety of livestock and wildlife asymptomatically, and these animals may serve as a reservoir of infection for humans^5^,^6^. Human *C. jejuni* infection can result in an asymptomatic carrier state, watery or bloody diarrhea, bacteremia, meningitis, or autoimmune neurological sequelae^7^,^8^. Despite the high prevalence of *C. jejuni* infection, its significant medical and economic consequences, and the availability of the *C. jejuni* genome sequence, the molecular and cellular mechanisms of campylobacteriosis pathogenesis are still poorly understood^9^. This is mainly due to the lack of robust experimental models that mimic the various phases of acute human infection.

Previous models include a ferret diarrheal model, a chick colonization model, and a colostrum-deprived piglet model^10^. However, the widespread use of these models, like other mammalian and avian models, has been limited by factors such as cost, ease of use, reproducibility, and ethics. Even though some mouse intestinal colonization models have recently become available, they are not suitable for cytokine studies because they were developed from cytokine-knockout mice^11^. Mice deficient in Single IgG Interleukin-1 related receptor (SIGIRR) were employed as an animal model for *C. jejuni* infection^12^. These studies have given us new insights into murine *Campylobacter* colonization, but the relationship between the observations in these models and human disease is unclear. Recently, infant rabbits have been used to study the molecular pathogenesis of other species-specify the organism and achieved good results^13^. Rabbits are more closely related to primates than rodents are in phylogeny and have far-reaching significance because they more closely resemble primates in anatomy and physiology^14^. In many instances, this leads to a more accurate modelling of human infections. In addition, the infant rabbit shares many advantages with rodents, such as small size, short gestation period, large litter size, ease of breeding and colony maintenance, and a relatively low purchase and housing costs.

In this study, the minimal dose of pathogenic *C. jejuni* strain NCTC 11168 required to establish clinical signs of disease in infant rabbits was determined, and a panel of multilocus sequence typed (MLST) *C. jejuni* field isolates were also screened for virulence. Different MLST groups were chosen to cover *C. jejuni* types detected in the main food-producing animals and in clinical samples. The pathogen primarily colonized the large intestine, where dramatic histopathologic and ultrastructural changes in the epithelium were observed. *C. jejuni* also induced intestinal inflammation in the rabbit model, the inflamed intestines had increased levels of IFN-γ, TNF-α, IL-1β, IL-2, IL-6, IL-8, and IL-22 gene expression. Acceptable animal models of infection are essential to characterization of putative bacterial virulence factors. Here we used the selective capture of transcribed sequences (SCOTS) approach, which has been used to screen virulence genes for many other species^15^,^16^,^17^,^18^,^19^,^20^,^21^,^22^,^23^,^24^, to identify *C. jejuni* genes preferentially expressed in the cecum of rabbits with acute *C. jejuni* infection. The identification of these genes will increase our understanding of the survival mechanism of the bacterium *in vivo* and its molecular pathogenesis.

## Results

### Clinical and histologic signs of disease after orogastric inoculation of *C. jejuni* into infant rabbits

**Figure 1:**
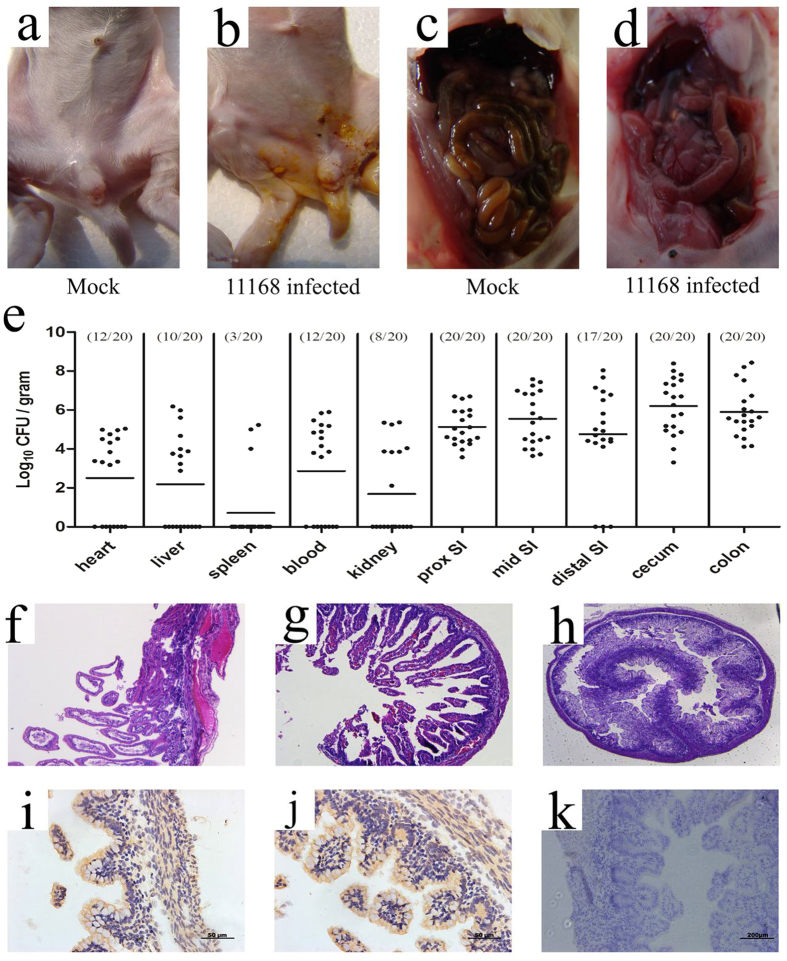
*C. jejuni* colonizes infant rabbits and induces destructive enteritis. Gross findings in infant rabbits inoculated with *C. jejuni* (**b**,**d**) or buffer (**a**,**c**). Rabbits exhibiting severe diarrhea in (**b)** and no diarrhea are detected in (**a**). The infected rabbits show the swollen, fluid-filled distended intestines (**d**) while the normal-appearing intestine is observed in (**c**). Recovery of *C. jejuni* (cfu g^−1^) from tissue homogenates of indicated organs and intestinal sections (prox, proximal and SI, small intestine) are displayed in (**e**). Points represent individual rabbits, bars represent geometric means. Representative sections of the intestines of infant rabbits 24 h after oral administration of *C. jejuni* strain NCTC 11168 are analyzed. Sections were either stained with hematoxylin and eosin (**f**–**h**, and **h** as control) or immunostained for *C. jejuni* (**i**–**k**, and **k** as control). Widespread edema and marked congestion of capillaries are observed in cross section of the cecum from an infant rabbit given oral *C. jejuni* (**f**,**g**), and no apparent pathological damage are observed in the uninfected control (**h**). The immunohistochemical stained *C. jejuni* (brown stain) can be seen in deep tissues as well as the paracellular junction and at the basolateral surface of the epithelium (**i**,**j**), while no positive staining are seen in the same cross section of the cecum from an uninfected control rabbit (**h**). Magnification, x100.

Previously, oral administration of *C. jejuni* to 6-day-old rabbits resulted in transient colonization but no evidence of disease^25^. Therefore, we used 24- to 36-h-old rabbits treated with cimetidine and observed reproducible diarrhea after experimental infection. Cimetidine is a histamine H_2_ receptor antagonist that transiently alleviates gastric acidity^26^. In almost all rabbits, inoculation with 1 × 10^9^ colony-forming units (cfu) of *C. jejuni* resulted in the release of loose, unformed, gelatinous stools, followed by yellow diarrheal fluid (Fig. 1b); the average time between infection and the development of diarrhea was 23.7 h. None of the rabbits in our experiments died, and the diarrhea spontaneously remitted by ~60 h after inoculation, thus lasting an average of 23.2 h. Campylobacteriosis is not a fatal infection in humans per se, and a considerable proportion of human cases of campylobacteriosis exhibit mild manifestations or remain asymptomatic^2^. Therefore, severe illness scores are not a prerequisite for the diarrheal model of *C. jejuni*. In most experiments described below, we used time points prior to 48 h to permit a variety of analyses at times when most rabbits exhibited disease.

The intestines of most (8/12) infected rabbits were swollen and red, and the small intestines, ceca, and proximal colons of infected rabbits were apparently distended and full of fluid (Fig. 1d). In contrast, mock-infected rabbits exhibited normal stool throughout the experiment and did not exhibit signs of illness (Fig. 1a,c).

Histological analyses revealed abnormalities in the distal small intestine as well as in the colon. There was widespread edema and marked congestion of capillaries in the villi of the cecum of infected rabbits compared with mock-infected control rabbits (compare Fig. 1f–h). Histological abnormalities were not very consistent in colonic sections from infected rabbits because submucosal edema and inflammatory cells were only apparent in some animals. Based on immunohistochemical staining, *C. jejuni* strain NCTC 11168 was found within the ceca of rabbits (Fig. 1i,j), suggesting that this strain is capable of invading intestinal tissues. Stained bacterial cells were observed in deep tissues as well as the paracellular junction and at the basolateral surface of the epithelium.

### Colonization of infant rabbit intestines by *C. jejuni*

**Figure 2:**
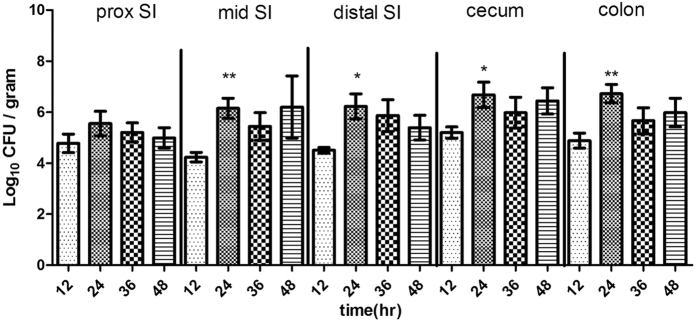
Numbers of *C. jejuni* recovered from homogenates of intestines. Proximal (prox), SI (small intestine). Values significantly greater than the values at 12 h postinfection are indicated by asterisks: *P < 0.05; **P < 0.01.

After oral inoculation of 2-day-old rabbits with *C. jejuni*, the bacterium could be cultured from the intestinal tract over the next 48 h (Fig. 2). For most subsequent experiments, 24 h postinoculation was chosen as the optimum time point to determine intestinal tract colonization by *C. jejuni* because the yield of *C. jejuni* was the highest and the standard deviation was the lowest at this time point. Following infection of infant rabbits, the intestinal tract appeared to be the primary target of *C. jejuni*. At 24 h postinfection, *C. jejuni* was isolated from 100% of cecum, colon, and jejunum samples, and the highest loads were approximately 10^6^ cfu g^−1^ (Fig. 1e). Ten-fold fewer cfu were recovered from the duodenum (100%) and ileum (85%). Additionally, *C. jejuni* was isolated from the spleen, liver, kidney, and cardiac blood of experimentally infected rabbits (Fig. 1e). Maximum isolation rates at 24 h postinfection were from cardiac blood (60%), followed by liver (50%), kidney (40%), and spleen (15%).

Tissue homogenates from diverse regions of the intestine were analyzed at various times postinfection to characterize the distribution and colonization dynamics of *C. jejuni* in the infant rabbit intestine. At 12 h postinoculation, <10% of the initial inoculum was recovered from the intestinal tract (Fig. 2), indicating that there was a considerable decline in the amount of organisms during passage through the stomach. By 24 h postinoculation, the number of *C. jejuni* cfu recovered from all regions of the intestine had increased by ~2 to 3 orders of magnitude (Fig. 2). At all time points, the cecum contained the highest number of *C. jejuni*, a finding consistent with analyses of human campylobacteriosis^2^. There were small decreases in the number of cfu recovered from each region of the intestine between 24 and 36 h postinoculation, a period characterized by secretory diarrhea in these animals, probably because diarrheal shedding restricted further bacterial accumulation. By the final time point, ~60 h postinoculation, *C. jejuni* could not be isolated from the small intestine. However, >1 × 10^5^ cfu per gram of tissue was routinely isolated from the large intestine, suggesting that *C. jejuni* can persist, if not multiply, in this environment. Overall, these results suggest that, after orogastric inoculation into infant rabbits, *C. jejuni* has the ability to survive and multiply within the intestinal tract.

### Difference in virulence in infant rabbits among *C. jejuni* MLST groups

Dose-response for diarrheal disease and intestinal colonization in rabbits were determined for *C. jejuni* strains NCTC 11168 and ATCC 33560 (Table 1). Both the severity of diarrhea and the degree of bacterial colonization were positively correlated with the challenge dose. The infectious dose needed to elicit both diarrhea and colonization was low (10^6^ cfu), thought as few as 800 cfu organisms can induce human volunteers diarrhoea^10^. Cecal colonization occurred when rabbits were orally inoculated with as little as 10^4^ cfu of *C. jejuni*. The two *C. jejuni* isolates varied in their virulence and their ability to colonize the intestine (Table 1). Diarrhea occurred when rabbits were orally inoculated with as little as 10^6^ cfu of *C. jejuni* NCTC 11168, in contrast, diarrhea was observed only after administering 10^10^ cfu of *C. jejuni* ATCC 33560.

**Figure 3:**
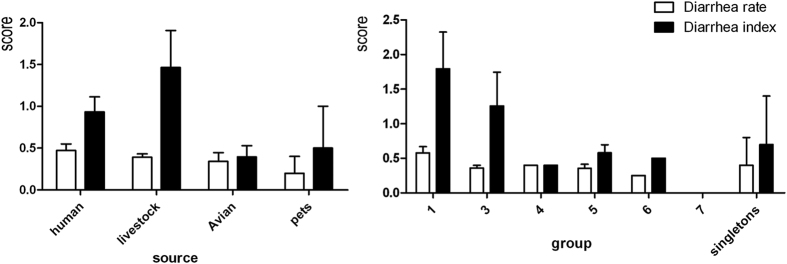


To investigate whether there was an association between MLST group and virulence, we recorded diarrhea rates and diarrhea indices following challenge with 22 *C. jejuni* strains belonging to different MLST groups using the dose of 10^9^ cfu (Table 2). Variation was observed both within and between MLST groups. Only one strain (PO09-1, isolated from a human source) produced a moderate diarrhea rate ≥80%. Strains from human and livestock sources showed greater virulence than those from avian sources. The ability of group 1 strains to cause disease was significantly different from that of other strains (Fig. 3).

### *C. jejuni* induces striking ultrastructural changes in the villus epithelium

**Figure 4:**
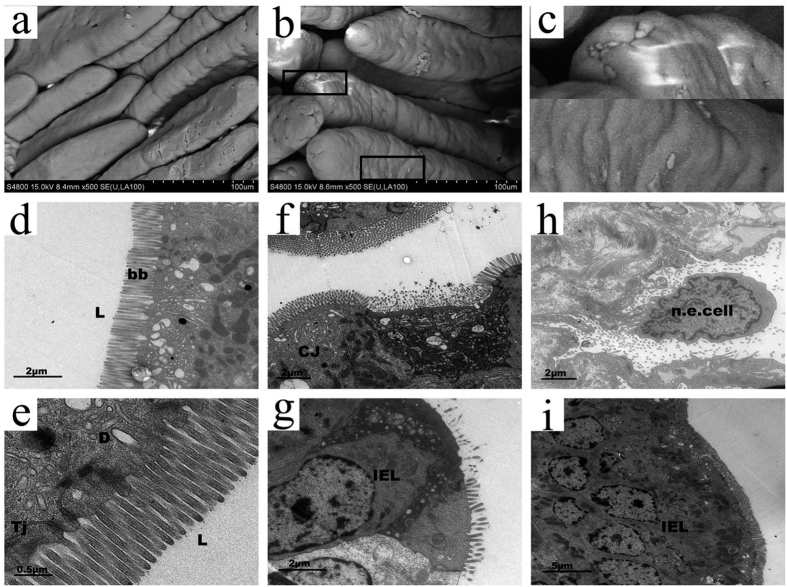
Scanning and transmission electron micrographs of the distal small intestine of rabbits inoculated with *C. jejuni*. Scanning electron micrograph of the small intestine of mock-infected (**a**) and *C. jejuni*-infected infant rabbit (**b**) at 24 h postinoculation. Boxed area of (**b**) is magnified in (**c**) to show the *C. jejuni* like bacteria colonizing the epithelial surface. Scale bar = 100 μm. The small intestine of *C. jejuni*-infected infant rabbit are also observed by transmission electron micrograph at 12 h (**d–e**), 24 h (**f–g**), 36 h (**h**) and 60 h (**i**) postinoculation. No *C. jejuni* are observed in lumen (L) and brush border (bb) remain intact at 12 h postinoculation (**d**), scale bar = 2 μm. Higher magnification image of (**d**) showing the tight junctions (Tj) and desmosomes remain intact (**e**), scale bar = 0.5 μm. Cluster of *C. jejuni* (CJ) locate in a cavity below the normal level of the surrounding epithelium when 24 h postinoculation (**f**), note the brush border of adjacent cells remain intact. Scale bar = 2 μm. At the same time, epithelium is destroyed and intraepithelial lymphocyte (IEL) infiltrating are observed (**g**), scale bar = 2 μm. *C. jejuni* locates at the base of a necrotic epithelial cell (n.e. cell), with more bacteria in the intestinal lumen at 36 h (**h**), scale bar = 2 μm. Following a severe inflammatory infiltrate of intraepithelial lymphocytes are observed at 60 h postinoculation (**i**), scale bar = 5 μm.

To further study how *C. jejuni* interacts with host epithelial cells, we visualized the epithelium in the small intestine of infected rabbits by scanning and transmission electron microscopy (EM). Clusters of attached bacteria were observed with scanning EM by 24 h postinfection, particularly near the villus tips (Fig. 4b, boxes); this phenomenon was not observed in samples from mock-infected rabbits (compare Fig. 4a,b). Transmission EM showed that *C. jejuni* did not induce any structural changes in the epithelium during the first 12 h after infection. *C. jejuni* was not observed by EM in the lumen, tight junctions, desmosomes, or brush border, which remained intact (Fig. 4d,e). However, *C. jejuni*-like bacteria were observed below the epithelial cell layer within cells of the lamina propria at 24 h postinfection. Partial destruction of the brush border, infiltration of intraepithelial lymphocytes, and intercellular swelling were also observed (Fig. 4f,g). The overall architecture of the epithelium was disorganized, and necrotic epithelial cells were present at 36 h postinfection (Fig. 4h). Severe infiltration of intraepithelial lymphocytes was observed at 60 h postinfection (Fig. 4i).

### *C. jejuni* induces intestinal immunopathology in infant rabbits

**Figure 5:**
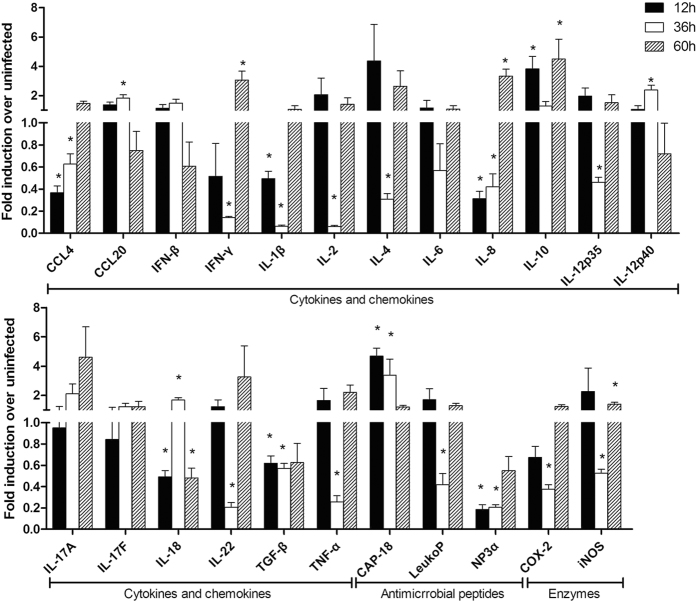
Changes in gene expression in the jejunum of rabbits infected with *C. jejuni*. The fold induction in gene expression over uninfected samples is given as the median of six samples obtained from two rabbits in three independent experiments. Black bars, white bars, and hatched bars represent 12 h, 36 h, and 60 h postinfection, respectively (*P < 0.05, Wilcoxon signed rank test).

We assayed the expression of genes for three chemokines (interleukin 8 [IL-8], chemokine [C-C motif] ligand [CCL] 4, and CCL20), 15 cytokines (IL-1β, IL-2, IL-4, IL-6, IL-10, IL-12 p35, IL-12/IL-23 p40, IL-17A, IL-17F, IL-18, IL-22, interferon [IFN] β, IFN-γ, transforming growth factor β, and tumor necrosis factor [TNF] α), three antimicrobials (leukocyte protein p15, neutrophil defensin, and the cathelicidin CAP-18), and two enzymes (inducible nitric oxide synthase and cyclooxygenase-2). This set of 23 genes exhibited a wide range of basal expression levels in infant rabbits from 12 to 60 h postinfection (Fig. 5). At the early time point of infection (12 h), there was a minor increase in the expression of genes for the anti-inflammatory cytokine IL-10 (3.837-fold) and the early antimicrobial CAP-18 (4.69-fold) compared with controls, while expression of the IL-4 gene was strongly induced in some but not all samples. After 36 h of infection, *C. jejuni* had induced a slight increase in the expression of some chemokine and cytokine genes, namely those for IL-12 p40 (2.389-fold), CCL20 (1.819-fold), and IL-18 (1.682-fold), while the expression of genes for many proinflammatory cytokines (IL-6, TNF-α, IL-1β, IFN-γ) was strongly reduced (0.06–0.67-fold). After 60 h of infection, at the more acute phase, the bacteria had induced a substantial increase in the expression of genes for most proinflammatory cytokines, including IFN-γ, IL-1β, IL-2, IL-6, IL-8, IL-22, and TNF-α, as well as genes for IL-10 and IL-4. The genes for the antimicrobials and enzymes, except CAP-18, were also up-regulated at this time point.

### Nucleotide sequence analysis of cDNA clones obtained by SCOTS

Three rounds of differential hybridization, of the normalized cDNAs from *C. jejuni* grown *in vivo*, to the biotinylated genomic DNA fragments, were performed to screen the genes differentially expressed in the rabbit cecum. The cecum-specific cDNAs were ligated into the pMD20-T vector (TaKaRa Biotech) and amplified by PCR. A total of nine differentially expressed genes were identified. These nine genes were divided into four functional groups: metabolism, cell surface, replication, and stress response (Table 3).

**Figure 6:**
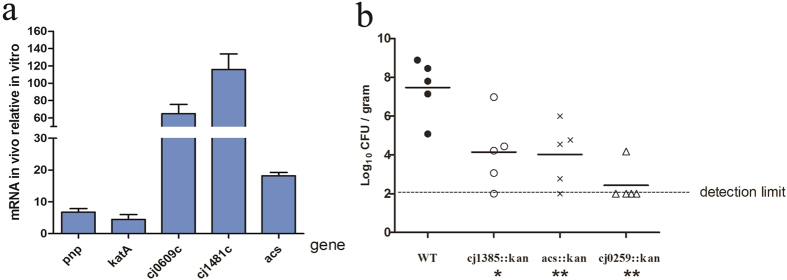
Analysis of genes differentially expressed by *C. jejuni* in the cecum of rabbits and recovery of *C. jejuni* strains from cecum. (**a**) Real-time RT-PCR analysis of the differentially expressed genes. (**b**) Recovery of wild-type (WT) or mutant *C. jejuni* (cfu) from cecum homogenates 12 h postinfection. Each symbol represents the value for one rabbit. Horizontal bars represent median values. Median values significantly lower than those found in wild-type infection are indicated by asterisks below the groups on the graph (*P < 0.05; **P < 0.01).

**Figure 7:**
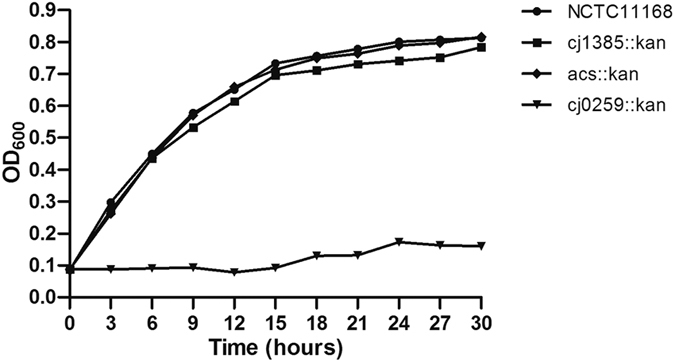
*In vitro* growth curves for *C. jejuni* NCTC 11168 and *C. jejuni* 1385, acs and 0259 mutants. Bacterial cultures were adjusted to an optical density at 600 nm (OD_600_) of 0.08 and grown in MH broth under microaerophilic conditions with shaking at 42 °C. The relationship of OD_600_ to viable count was equivalent for all strains examined.

To investigate the genes differentially expressed by *C. jejuni* strain NCTC 11168 in infected rabbit ceca, five genes (*pnp*, *katA*, *cj0609c*, *cj1481c*, and *acs*) were randomly selected and analyzed by qRT-PCR. The expression levels of all five genes were up-regulated in infected rabbit ceca compared with *in vitro* cultures. Compared with the housekeeping gene *cj0402*, used as an internal control, the changes in gene expression ranged from 4.49 fold to 116 fold (Fig. 6a). To explore the pathological effects of these genes within our model system, we constructed three *C. jejuni* mutant strains. Infant rabbits inoculated with wild-type *C. jejuni* and *cj1385*, *acs*, and *cj0259* mutants subsequently acquired diarrhea index scores of 1.73, 2.67, 1.67 and 1.00, respectively. All the mutants seemed to be required for *C. jejuni* colonization of infant rabbit intestine. The number of bacteria recovered from the cecum of rabbits infected with the *cj0259* mutant was more than 4 orders of magnitude lower than the number recovered from rabbits infected with wild type *C. jejuni* (Fig. 6b), perhaps due to unspecified *cj0259* mutant growth defects *in vitro* (Fig. 7).

## Discussion

Here, we developed a simple nonsurgical animal model of campylobacteriosis. Cimetidine-treated 2-day-old infant rabbits developed diarrhea within 48 h after oral inoculation of live *C. jejuni*. The clinical signs and pathological lesions produced in the infant rabbits mimicked those observed in humans and other large-animal models. *C. jejuni* multiplied in the intestines of rabbits, the cecum was the most consistently colonized site and contained the largest populations of *C. jejuni*, indicating its possible role in the pathogenesis of campylobacteriosis^27^, *C. jejuni* could also be isolated from the spleen and other organs of some rabbits, indicating that this pathogen is capable of systemic invasion. Dose-response experiment showed that while 10^10^ cfu of *C. jejuni* ATCC 33560 were required to cause diarrhea, 4 log fewer cfu of *C. jejuni* NCTC 11168 were needed, this was similar with previous studies in humans or monkeys^28^,^29^. With most *C. jejuni* strains tested in this study, a moderate diarrhea incidence of ≥80% was not achieved even at a dose of 10^9^ cfu, which was in contrast to intragastric administration of mice^11^,^30^. However, we did not confirm that all of the tested *C. jejuni* isolates are enteropathogenic. As the ability of the bacterium to cause bloody diarrhea is a bacterial attribute^31^,^32^,^33^, and symptoms caused by a single strain can vary among individuals^34^,^35^. So it is possible, and even probable, that many of the genome-sequenced strains have lost virulence during laboratory passage.

This study was also designed to determine whether *C. jejuni* phylogeny or strain-specific gene content was associated with a particular pathotype in the rabbits. The results of MLST analysis suggest a potential association of pathotype with phylogeny. A number of studies have sought to establish a link between MLST type and virulence^36^,^37^,^38^. Researchers used the model organism *Galleria mellonella* to screen 67 *C. jejuni* isolates belonging to different MLST types. Isolates belonging to sequence type (ST) 257 were the most virulent in *G. mellonella*, whereas those belonging to ST21 were the least virulent. In contrast, we found that isolates belonging to MLST group 1, which contains ST21 and ST206, were the most virulent in our rabbit model. Therefore, these results should be viewed with caution, as described above, many strains may have lost virulence during laboratory passage, and this outcome is especially likely for the sequenced strains and the type strain NCTC 11168, which have been studied extensively in the laboratory. Other strains may be capable of causing disease following adaptation to the rabbit GI tract. Therefore, the potential association of pathotype with phylogeny in MLST group 1 must be viewed as intriguing but in need of verification by further work using minimally passaged isolates.

*Campylobacter* infection is generally indistinguishable from acute gastrointestinal infections produced by other bacterial pathogens. So the primary task of this study was to establish an animal model and to document the severity as well as the time course of disease manifestations mimicking “classical” features of human campylobacteriosis. Scoring the diarrhea is clearly straightforward and easy to do and would also monitor the entire process of disease. Since historically pathology is a very important indicator of virulence which we had also heeded in previous research^13^,^39^. However, a characteristic of *C. jejuni* may be that pathologies are weakly correlated with the severity of diarrhea symptoms in the infant rabbit model. In our experience, rabbits displaying severe diarrhea sometimes did not show serious pathological damage. This phenomenon may be incidental, or may be due to the relatively weak pathogenicity of the bacterium itself. We are now trying to find a velogenic strain that can reproducibly induce pathological changes in infant rabbits. We have also attempted to construct a mutant whose virulence is dramatically reduced, in order to establish a detection system for pathological changes.

The infant rabbit model of *C. jejuni* infection should be useful in exploring the role of innate immunity. Previous studies suggested that *C. jejuni* may induce IL-4 and IL-10 to help control the infection^40^, and reduce IL-6, IL-8, TNF-α, IL-1β, and IFN-γ to alleviate the inflammation. Thus, these cytokines seem to be involved in controlling the inflammatory response to the infection, and protecting against further intestinal injury^41^. In our rabbit model, *C. jejuni* induced intestinal inflammation and increased the expression of IFN-γ, TNF-α, IL-1β, IL-2, IL-6, IL-8, and IL-22. The induction of TNF-α and IFN-γ indicates activation of macrophages and type 1 helper T cells and is related to accumulation of polymorphonuclear cells in the intestines of infected rabbits. Infection of human intestinal epithelium with *Campylobacter* results in activation of nuclear factor κB, which is needed for the induction of proinflammatory cytokine genes such as IL-8^42^. IL-6 is an atypical cytokine with dual functions. The significance of IL-6 as a pro-inflammatory cytokine is suggested by a concomitant increase in the levels of IL-1β. IL-1β and TNF-α are known to stimulate the production of IL-6. Therefore, it is not unlikely that the production of IL-6 is a mechanism that perpetuates the inflammatory response to *C. jejuni* infection. Previous studies have showed that an enterotoxigenic strain of *C. jejuni* could induce the production of IL-2, while it lacked the ability to stimulate the production of macrophage-derived cytokines^43^. Similarly, TNF-α, IL-1β, IL-6, and IL-8 were also found to be the major components of the immune response to orogastric infection with other species^39^,^44^. Our rabbit model has several limitations. One is that, this model is not suitable for vaccine efficacy study because active immunization is not possible and immune system is not so much developed.

Gene expression in *C. jejuni* strain NCTC 11168 after infection of experimental animals has been studied in other researchs^45^,^46^. To further utilization our model, putative virulence factors were screened to explore the pathogenesis of *C. jejuni*. A total of nine putative virulence genes were found by SCOTS, as in the previous studies, the *katA* gene was found to be differentially expressed in *C. jejuni*. This gene encodes catalase, an oxidative stress defense protein that may be associated with iron effect and stress response, which was also identified by SCOTS for other species^21^,^47^. The polynucleotide phosphorylase (Pnp) was found, in a previous study using signature-tagged mutagenesis (STM), a *pnp* mutant was identified and found to be attenuated in mice, the *pnp* gene was also identified in rabbit liver using SCOTS^19^,^48^. In *C. jejuni*, inactivation of the *pnp* gene significantly reduced the ability of the bactrium to adhere to and invade HT-29 cells and exhibited decreased swimming ability and chick colonization^49^. Long-term survival of *C. jejuni* at low temperatures is also dependent on Pnp activity^50^. The other three genes we identified seemed to be required for *C. jejuni* colonization, but did not affect pathogenicity. *Cj0259* is critical for *C. jejuni* growth, as inactivation of *Cj0259* causes a significant growth defect in this bacterium. However, this growth defect may be only one of the factors responsible for the decreased virulence phenotype, as *Cj1385* and *acs*, which also decrease the colonization ability of *C. jejuni,* were not involved in *C. jejuni* normal growth. Therefore, future work must examine these genes by scoring diarrhea and/or other pathologies, or cytokine response, to see if the mutations affect *C. jejuni* invasion of tissues.

Collectively, our findings suggest that infant rabbits may be a useful experimental model to study the mechanisms underlying the pathogenesis of *C. jejuni*-induced intestinal disease, but also have the potential to explore the pathogenic mechanism of other related pathogens. Development of this infant rabbit model based on pathogen infection will enable a variety of future studies. For example, this model could be used to study the roles of cytokines in the pathogenesis of bacterium-induced disease. Furthermore, the model could be used to screen clinical isolates for virulence to help elucidate the connection between the extensive and well-described genetic variation of the pathogen, and the spectrum of symptoms and adverse clinical sequelae associated with this pathogen. The model could also be used to examine aspects of general evolutionary interactions between pathogens and their hosts, increasing our understanding of such interactions is a matter of pressing concern for global public health.

## Methods

### Ethics statement

All animal trials in this study were carried out in strict accordance with the Regulations for the Administration of Affairs Concerning Experimental Animals approved by the State Council of the People’s Republic of China (11-14-1988). The animal experimental design and protocols were approved by the Institutional Animal Care and Use Committee (IACUC) of Yangzhou University (Permit Number: 2012-62).

### Bacterial strains, media, and growth conditions

The key *C. jejuni* strains used in this study are listed in Tables 1 and 2. Strains NCTC 11168 and ATCC 33560 were gifts of Dr. Wanbang Sun, Zunyi Medical College, China. *C. jejuni* strains to be inoculated into rabbits were grown on *Campylobacter* blood-free selective medium (CCDA) plates (Oxoid, Basingstoke, Hampshire, England) containing 5% defibrinated sheep’s blood under an atmosphere of 5% O_2_, 10% CO_2_, and 85% N_2_ at 42 °C. *C. jejuni* was isolated from rabbit tissues by streaking on selective CCDA containing 5% defibrinated sheep’s blood, 10 mg mL^−1^ amphotericin B, and 20 mg mL^−1^ cefoperazone (all antibiotics from Sigma-Aldrich, St. Louis, MO, USA). Plates were incubated in closed containers at 42 °C under microaerobic conditions for 24 h before counting colony-forming units. Cultures were verified using a *C. jejuni*-specific *mapA* and 16S rRNA gene PCR assay as previously described^51^.

*E. coli* DH5α was used as the host strain for the construction and maintenance of plasmids containing the 16S and 23S rRNA genes and all SCOTS clones in the pMD20-T vector (TaKaRa, Dalian, China). *E. coli* was routinely grown at 37 °C in Luria-Bertani (LB) medium (Oxoid, Basingstoke, England) supplemented with ampicillin (50 μg mL^−1^), isopropyl-β-d-thiogalactoside (100 μg mL^−1^), and X-gal (200 μg mL^−1^) when required.

For gene expression studies, *C. jejuni* NCTC 11168 was grown to mid-exponential phase (approximately 24 h) on CCDA plates at 42 °C under microaerobic conditions, harvested, and centrifuged (12,000 g, 4 °C, 5 min). Cell pellets were resuspended in RNAlater (Ambion) and were then used for RNA extraction.

### Infection of rabbits with *C. jejuni*

Litters of newborn New Zealand White rabbits with the lactating doe were acquired from a commercial breeder (Jinling Farm, Nanjing, Jiangsu, China). The following day, the infant rabbits received cimetidine (50 mg kg^−1^ via intraperitoneal injection) 3 h prior to orogastric inoculation with either 1 × 10^9^ cfu of *C. jejuni* strain suspended in sodium bicarbonate solution (2.5 g in 100 mL, pH 9) or sodium bicarbonate solution alone (negative control) using a size 4 French catheter (Arrow International, Reading, PA, USA). To prepare the inocula, we harvested bacteria from agar surfaces using cell scrapers (BD Falcon, BD Biosciences, Bedford, MA, USA) and resuspended the bacteria in sodium bicarbonate solution to an optical density at 600 nm of 0.8 to 1.0 (approximate concentration, 5 × 10^9^ cfu mL^−1^) as previously described^13^. The purity and motility of cultures were verified by light microscopy and Gram stain, and bacterial counts (cfu per milliliter) were determined by serial dilution.

Following inoculation, the infant rabbits were monitored twice per day for clinical signs of illness. Diarrhea disease was scored as follows: Grade 0, none, normal stool; Grade 1, mild, soft to loose stool, no adherent fecal material on fur; Grade 2, moderate, loose stool or mixed with mucus, liquid fecal material staining or adhering to fur; Grade 3, severe, frequent loose or projectile watery stool with mucus, fecal material staining large portions of the perineum, hind legs, and tail. Diarrhea rate is the number of rabbits with diarrhea divided by the total number of rabbits. Diarrhea index is the sum of disease grades divided by the total number of rabbits. Rabbits exhibiting severe clinical signs were euthanized and necropsied promptly.

For the determination of dose response for different isolates of *C. jejuni*, 52 2-day-old rabbits were allocated into 13 treatment groups (four rabbits per group) and housed in separate battery brooders. The 13 treatment groups were as follows: control rabbits; rabbits inoculated with doses of *C. jejuni* NCTC 11168 from 10^10^ to 10^2^ cfu; and rabbits inoculated with doses of *C. jejuni* ATCC 33560 of 10^10^ cfu, 10^8^ cfu, or 10^6^ cfu. The amounts of inoculum used in the dose-response experiments are given in Table 1; doses were serially diluted for cfu determination.

### Necropsy and sampling procedures

In most experiments, rabbits were euthanized by CO_2_ overdose at fixed times after infection (i.e., 12, 24, 36, or 48 h postinfection). Immediately after euthanasia, a blood sample was obtained by cardiac puncture using a 25-gauge needle on a 1-mL tuberculin syringe containing 0.1 mL of 3.8% sodium citrate. Trained individuals noted gross pathological changes in all portions of the GI tract. The intestinal tract from the duodenum to the rectum was removed and processed for histologic and microscopic analyses, RNA extraction, and enumeration of *C. jejuni* (cfu per gram of tissue). For some rabbits, the internal organs including the heart, spleen, kidney, and liver were also collected, homogenized, and plated on selective media to check for systemic spread of *C. jejuni*.

Rabbits that showed typical clinical signs of diarrhea at 24 h postinfection were euthanized humanely. Cecum samples containing 10^6^–10^8^ cfu per gram of tissue were obtained from four rabbits and used for SCOTS.

### Intestinal colonization and histopathology

At selected time points postinoculation, a subset of rabbits were euthanized, and their GI tracts were aseptically removed. Sections from the duodenum, small intestine, large intestine, and cecum were harvested following gentle extrusion of the lumenal contents. The sections were weighed and homogenized in sterile phosphate-buffered saline. Homogenates were serially diluted, plated on selective medium, and incubated for 36 to 48 h. *C. jejuni* recovery is expressed as cfu per gram of tissue.

Cecum samples were immediately fixed in 10% neutral buffered formalin and embedded in paraffin. Sections (5 μm) were stained with hematoxylin and eosin and examined by light microscopy. For immunohistochemical staining for *C. jejuni*, sections were permeabilized with 0.1% Triton X-100 for 15 min, treated with 3.3% H_2_O_2_ for 15 min, and washed. Samples were blocked for 30 min with 5% bovine serum albumin and incubated for 1 h with an in-house mouse polyclonal antiserum against *C. jejuni* or without serum as a control. Samples were then incubated with horseradish peroxidase-conjugated goat anti-mouse IgG (1:5000; Sigma) for 1 h, developed with 3-amino-9-ethylcarbazole, counterstained with hematoxylin, and mounted with aqueous mounting medium.

### Electron microscopy

Intestinal tissues were fixed for 3 h in 0.1 M cacodylate buffer containing 2.5% glutaraldehyde and 2% paraformaldehyde, pH 7.4. All buffer reagents were purchased from the Test Center of Yangzhou University, Yangzhou, Jiangsu. The tissues were postfixed in 1% osmium tetroxide, dehydrated, and prepared using standard procedures for transmission or scanning electron microscopy as described previously^39^,^52^. The samples were examined with an S-4800 scanning electron microscope and a Philips Tecnai 12 transmission electron microscope.

### RNA isolation and cDNA synthesis

For RNA extraction, 1 cm long tissue samples were immediately submerged in 4 mL of TRIzol Reagent (Invitrogen), homogenized for 1 min with a tissue homogenizer, and stored at −80 °C until further processing. A 1.2 mL aliquot of each sample was centrifuged at 4 °C for 15 min at 12,000 × *g* to remove debris and DNA. Then, 1 mL of supernatant was mixed with 200 mL of chloroform, shaken for 15 s, incubated at room temperature for 2–3 min, and centrifuged for 10 min at 12,000 × *g* at 4 °C. RNA was precipitated by adding 500 mL of the aqueous phase to an equal volume of isopropanol and centrifuging at 14,000 × *g* at room temperature for 10 min. RNA was washed with 75% ethanol, centrifuged at 14,000 × *g* at 4 °C for 10 min, dried, and resuspended in 60 μL of diethylpyrocarbonate-treated water (Ambion).

RNA samples were treated with DNase I (MBI Fermentas) and evaluated by gel electrophoresis before cDNA synthesis. For cytokine gene expression analysis, cDNA was synthesized using SuperScript II reverse transcriptase (Invitrogen) as recommended by the manufacturer and as previously described^14^, using RNA from jejunum samples. For SCOTS, cDNA was synthesized with random primers as previously described^19^, using RNA from cecum samples. cDNA was stored at −20 °C.

### SCOTS

The plasmids and primers used in this study are listed in Supplementary Table 1. Genomic DNA from *C. jejuni* strain NCTC 11168 was photobiotinylated as described previously^23^. For each round of SCOTS, 3 μg cDNA samples were denatured at 98 °C for 3 min, normalized by self-hybridization, and subsequently hybridized at 65 °C for 24 h with 0.6 μg of photobiotinylated *C. jejuni* strain NCTC 11168 genomic DNA that had been previously blocked with 5 μg of 16S and 23S rDNA. The hybridized cDNAs were captured by streptavidin-coupled magnetic beads (Dynal M280, Invitrogen) according to the manufacturer’s instructions. After elution, the cDNAs were re-amplified by PCR using the primer SCOTS-01. For each growth condition, 10 samples of the total cDNA mixture were captured separately by hybridization in parallel reactions in the first round of SCOTS, and the 10 amplified cDNA preparations were combined for two subsequent rounds. The final captured cDNAs were cloned into the pMD20-T vector (TaKaRa), and white clones on X-gal plates were sequenced using the standard Sanger method. Database searches and DNA and protein similarity comparisons were carried out using the BLAST algorithm from the National Center for Biotechnology Information at the National Library of Medicine ( http://www.ncbi.nlm.nih.gov/BLAST/Blast.cgi).

### Quantitative real-time PCR

Quantitative real-time reverse transcription PCR (qRT-PCR) was performed in a final volume of 20 μL containing 2.0 μL of cDNA, 16.4 μL of SYBR Premix Ex Taq II (TaKaRa), 10 μM forward primer, 10 μM reverse primer, and diethylpyrocarbonate-treated water. qRT-PCR was performed using a GeneAmp 7500 thermocycler (Applied Biosystems, Carlsbad, CA, USA) with the following protocol: 2 min at 50 °C followed by 40 cycles of denaturation at 95 °C for 30 s and annealing at 60 °C for 34 s. The specific oligonucleotide primers used to amplify rabbit cytokine cDNA sequences have been described previously^14^. The GAPDH gene was used as a control, and all cytokine values were normalized to the GAPDH value using the ΔΔC_T_ method as previously described^26^.

After qRT-PCR analysis of *C. jejuni* RNA derived from *in vivo* and *in vitro* cultures, five differentially expressed genes were selected and their expression levels relative to the housekeeping gene *cj0402* were determined in triplicate^53^. The relative expression level was calculated using the ΔΔC_T_ method.

### Creation of *C. jejuni* mutant

*C. jejuni* mutant strains used in this study were constructed by insertional inactivation, and the primers used for mutant construction are listed in Supplementary Table 2. Briefly, the target genes for mutagenesis were amplified form NCTC 11168 genomic DNA and ligated into the plasmid pMD-19T Simple (TaKaRa, Dalian, China). The kanamycin resistance cassette (*kan*^*r*^) was amplified from pRY107 using primers with a unique enzyme site included in the coding region of the target gene. All of the replicons were verified by sequencing, and the *kan*^*r*^product was then inserted into the target gene to complete construction of the suicide plasmid. The suicide plasmid was then electroporated into *C. jejuni* competent cells, and the mutants were selected on CCDA agar containing kanamycin 50 mg mL^−1^.

### Statistical analysis

Bacterial counts (after log transformation) were analyzed using one-way analysis of variance and Bonferroni’s test for multiple comparisons (Prism, GraphPad Software, San Diego, CA, USA). The fold induction of gene expression was assessed using the Wilcoxon signed rank test (GraphPad Prism).

## Additional Information

**How to cite this article**: Shang, Y. *et al*. Insights into *Campylobacter jejuni* colonization and enteritis using a novel infant rabbit model. *Sci. Rep.* **6**, 28737; doi: 10.1038/srep28737 (2016).

## Supplementary Material

Supplementary Information

## Figures and Tables

**Table 1:** Dose response for disease and intestinal colonization of rabbits by *C. jejuni* strains NCTC 11168 and ATCC 33560.

***C. jejuni*** **isolates**	**Dose of inocula (cfu)**^a^	**Diarrhea index**	**No. of** ***C. jejuni*****-positive rabbit/total no. of rabbits by PCR**^b^			**Colonization**^c^		
			**jejunum**	**cecum**	**colon**	**jejunum**	**cecum**	**colon**
NCTC 11168	10^10^	1.61	4/4	4/4	4/4	5.08 ± 0.25	6.27 ± 0.23	6.04 ± 0.34
	10^9^	1.73	4/4	4/4	4/4	5.30 ± 0.13	6.62 ± 0.21	6.09 ± 0.87
	10^8^	0.83	3/4	4/4	4/4	2.62 ± 2.27	4.55 ± 0.17	3.51 ± 0.91
	10^7^	0.4	2/4	4/4	3/4	1.31 ± 1.18	2.64 ± 0.48	2.64 ± 0.84
	10^6^	0.4	2/4	4/4	4/4	0.13 ± 0.22	2.79 ± 0.14	2.29 ± 0.43
	10^5^	0	2/4	2/4	2/4	0 ± 0	0.78 ± 1.11	0.61 ± 0.86
	10^4^	0	1/4	3/4	3/4	0 ± 0	1.22 ± 0.67	1.43 ± 0.48
	10^3^	0	0/4	2/4	1/4	0 ± 0	0 ± 0	0 ± 0
	10^2^	0	1/4	2/4	1/4	0 ± 0	0 ± 0	0 ± 0
ATCC 33560	10^10^	0.35	2/4	4/4	3/4	1.80 ± 1.56	4.00 ± 0.41	3.52 ± 0.30
	10^8^	0	2/4	4/4	1/4	1.19 ± 1.03	3.25 ± 0.60	0.74 ± 1.28
	10^6^	0	1/4	2/4	2/4	0.84 ± 1.46	2.13 ± 1.84	2.22 ± 1.93

^a^Rabbits were euthanized at 24 h postinoculation.

^b^All control animals were negative for *C. jejuni* by culture and PCR.

^c^Mean ± SD (n = 3) log_10_ CFU/g.

**Table 2:** Pathogenicity evaluation of different *Campylobacter jejuni* isolates.

**Group**	***C. jejuni*** **isolates (total no. tested)**	**MLST type (clonal complex)**	**Source**	**Diarrhea rate (100%)**	**Diarrhea index**
1	NCTC 11168 (12)	43 (21)	human	66.7	1.58
	LM-10 (6)	760 (21)	chicken	66.7	1
	TZDN-8 (5)	572 (206)	cattle	40	2.8
3	PO-53-1 (7)	3652 (22)	human	28.6	0.57
	DN-4-8 (5)	42 (42)	cattle	40	2.2
	DJDN-39 (5)	22 (22)	cattle	40	1
4	PO-13-9 (5)	137 (45)	human	40	0.4
5	POC0611-10 (4)	4246 (257)	human	50	1
	SO-38-6 (4)	2517 (353)	human	25	1
	SO-9-3 (5)	4332 (443)	human	20	0.2
	CW0903-2 (5)	4265 (52)	dog	40	1
	SQDN-14 (4)	2145 (574)	cattle	25	0.5
	DJDN-15 (6)	607 (607)	cattle	50	0.83
	QMJ-94 (3)	354 (354)	chicken	66.7	0.67
	d-1-5 (5)	2895 (574)	chicken	40	0.6
	ALM-52 (3)	4345 (460)	chicken	0	0
	SG0811-1 (5)	4259 (345)	goose	40	0.4
6	JDSXD0904-4 (4)	4252 (692)	duck	25	0.5
7	LHG0901-5 (4)	1269 (1034)	goose	0	0
Singletons	CW0803-3 (4)	4260 (UA)	dog	0	0
	PO09-1 (5)	2123 (362)	human	80	1.4
–	SO0707-4 (6)	–	human	66.7	1.33

**Table 3:** Genes expressed by *C. jejuni* strain NCTC 11168 during rabbit infection, as determined by SCOTS.

**Category**	**Clone**	**Locus name**	**Predicted fuction or property**
Metabolism	1102-1	*pnp, cj1253*	polyribonucleotide nucleotidyltransferase
	1102-2	*acs, cj1537c*	acetyl-coenzyme A synthetase
	1103-3	*pyrC, cj0259*	dihydroorotase
Cell surface	1105-3	*pglC, cj1124c*	GalNAc transferase
	1105-8	*cj1438c*	putative sugar transferase
	1105-11	*cj0375*	putative lipoprotein
Replication	1102-6	*recB, cj1481c*	putative helicase
	1102-7	*cj0609c*	possible periplasmic protein
Stress response	1102-25	*katA, cj1385*	catalase
