# Supplementary material for: Insights into Campylobacter jejuni colonization and enteritis using a novel infant rabbit model
Source: Sci Rep. 2016 Jun 30;6:28737. doi: 10.1038/srep28737 (PMC4928045; doi:10.1038/srep28737)
Supplement: Supplementary Information [file srep28737-s1.pdf]

Insights into *Campylobacter jejuni* colonization and enteritis using a novel infant rabbit model

Yuwei Shang[1,*], Fangzhe Ren[1,*], Zhaojun Song[1], Qiuchun Li[1], Xiaohui Zhou[2], Xiaobo Wang[3], Zhonglan Xu[4], Guangyu Bao[5], Ting Wan[1], Tianyao Lei[1], Nan Wang[1], Xin-an Jiao[1], Jinlin Huang[1]

[1] Jiangsu Key Lab of Zoonosis, Jiangsu Co-Innovation Center for Prevention and Control of Important Animal Infectious Diseases and Zoonoses, Yangzhou University, Yangzhou 225009, China. [2] Department of Pathobiology and Veterinary Science, the University of Connecticut, Storrs 06269, United States of America. [3] College of Veterinary Medicine, Yangzhou University, Yangzhou 225009, China. [4] Yangzhou Maternity and Infant Hospital, Yangzhou 225001, China. [5] Yangzhou First People's Hospital, Yangzhou 225004, China. * These authors contributed equally to this work. Correspondence and requests for materials should be addressed to J.H. (email: jinlin@yzu.edu.cn)

Supplementary Table 1. Characteristics of plasmids and primers in SCOTS study

plasmids or primers	Characteristics and/or sequences	Source/reference
Plasmid		
pMD20-T	Cloning vector, Amp^R	TAKARA
pMDCJ16S	pMD20-T containing the 16SrRNA gene sequence (1466 bp) of *C. jejuni* 11168	This work
pMDCJ23S	pMD20-T containing the 23SrRNA gene sequence (2724 bp) of *C. jejuni* 11168	This work
Primers		
SCOTS-N6-01	5'-GCCGGTCGACTGCAGAATTC-N6-3'	[1]
SCOTS-N6-02	5'-CTACGCATGCTCGAGGTACC-N6-3'	[1]
SCOTS-01	5'-GCCGGTCGACTGCAGAATTC-3'	[1]
SCOTS-02	5-CTACGCATGCTCGAGGTACC -3'	[1]
16srrn-F	5'-GGATCCCTGGCTCAGAGTGAACGC-3'	This work
16srrn-R	5'-GGTACCTACGGTTACCTTGTTACGAC-3'	This work
23srrn-F	5'-GAATTCCGAATGGTGGATGCCTTGA-3'	This work

23ssrn-R	5’-GGTACCACGCTGCGATGCTCTTGG-3’	This work
cj0402-F	5’-CGATGGAACGGATAATCACC-3’ amplifies *cj0402* gene for qRT-PCR	[2]
cj0402-R	5’-AATACCTGCATTTCCAAGAGC-3’	[3]
pnp-F	5’-TACCTCTAACCGTCCAA-3’ amplifies *pnp* gene for qRT-PCR	This work
pnp-R	5’-ATCAATAATGCGTGCTG-3’	This work
katA-F	5’-AAGGCGAAGTTACTCCA-3’ amplifies *katA* gene for qRT-PCR	This work
katA-R	5’-TTCCTACCAAGTCCCAG-3’	This work
cj0609c-F	5’-TTTCTTTCCGCGTGTCATA-3’ amplifies *cj0609c* gene for qRT-PCR	This work
cj0609c-R	5’-TTATGTCCAGCATTAGCAG-3’	This work
cj1481c-F	5’-CCATATCTGCATCGCTA-3’ amplifies *cj1481c* gene for qRT-PCR	This work
cj1481c-R	5’-TTGTGCGTATTGTGGAGTC-3’	This work
acs-F	5’-AGTCATTGTTTGCTTTTGTGG-3’ amplifies *acs* gene for qRT-PCR	This work
acs-R	5’-GATTTCTTCGCCTCTTGCT-3’	This work

References

1. Li, W., Liu, L., Chen, H. & Zhou, R. Identification of *Streptococcus suis* genes preferentially expressed under iron starvation by selective capture of transcribed sequences. *FEMS Microbiol. Lett.* **292**, 123-133 (2009).
2. Malik-Kale, P., Parker, C. T. & Konkel, M. E. Culture of *Campylobacter jejuni* with sodium deoxycholate induces virulence gene expression. *J Bacteriol.* **190** 2286-2297 (2008).
3. Schlech, W. F. 3rd, Chase, D. P. & Badley, A. A model of food-borne *Listeria monocytogenes* infection in the Sprague-Dawley rat using gastric inoculation: development and effect of gastric acidity on infective dose. *Int. J. Food Microbiol.* **18**, 15-24 (1993).

Supplementary Table 2. primers used for C.jejuni mutant construction

Primers	Sequence [a]	Source
cj1385-F	5'- CGC<u>GGATCC</u>TGACTAACGATTTTGGA -3' (*Bam*HⅠ)	This work
cj1385-R	5'- CGC<u>GAGCTC</u>GTCTTCTTTTGGGCTAT -3' (*Sac*Ⅰ)	This work
acs-F	5'- CGC<u>GGATCC</u>TTTCTCGTAATGCTCGTA -3' (*Bam*HⅠ)	This work
acs-R	5- CGC<u>GAGCTC</u>ATTCTTATGGCAGTTGG -3' (*Sac*Ⅰ)	This work
cj0259-F	5'-CGC<u>GGATCC</u>GGCATGAAAAGGCAAAA -3'(*Bam*HⅠ)	This work
cj0259-R	5'- CGC<u>GAGCTC</u>CACAACCGCAACACTCT -3' (*Sac*Ⅰ)	This work
kan(*Hin*d Ⅲ)-F	5'- CCC<u>AAGCTT</u>CGCTTATCAATATATCTATAGAATG -3' (*Hin*d Ⅲ)	This work
kan(*Hin*d Ⅲ)-R	5'- CCC<u>AAGCTT</u>GATAATGCTAAGACAATCACTAAA -3' (*Hin*d Ⅲ)	This work
kan(*Bgl* II)-F	5'- GGA<u>AGATCT</u>CGCTTATCAATATATCTATAGAATG -3' (*Bgl* II)	This work
kan(*Bgl* II)-R	5'- GGA<u>AGATCT</u>GATAATGCTAAGACAATCACTAAA -3' (*Bgl* II)	This work

[a] Restriction sites are underlined in the primer sequence.
